# Supplementary figures and images for: Pacemaker translocations and power laws in 2D stem cell-derived cardiomyocyte cultures
Source: PLoS One. 2022 Mar 14;17(3):e0263976. doi: 10.1371/journal.pone.0263976 (PMC8920264; doi:10.1371/journal.pone.0263976)

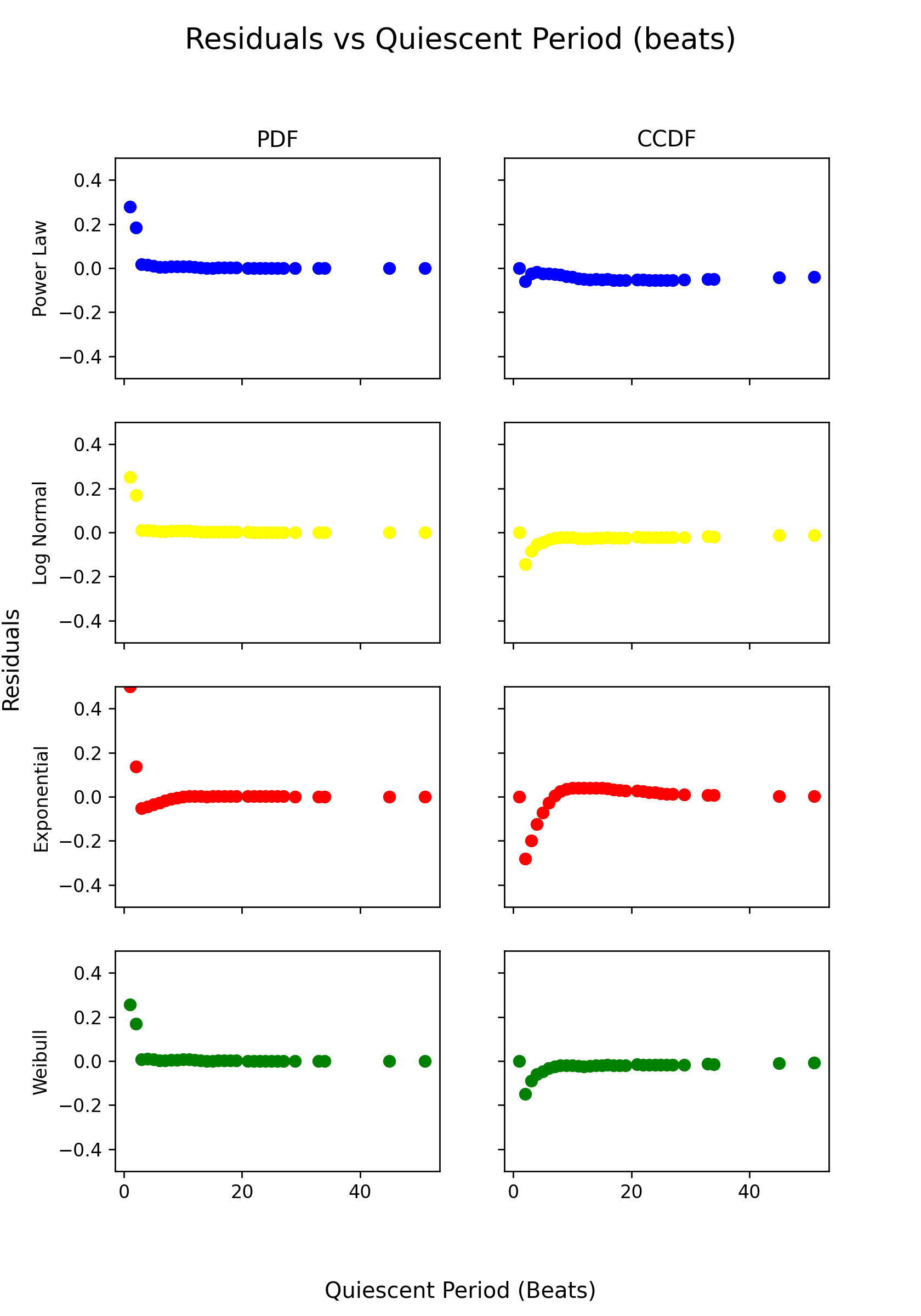

Supplement: S1 Fig — Each residual plot shows the difference between the real data values (empirical PDF or CCDF) and the model values (distribution PDF or CCDF). These residual values are plotted against the quiescent period as measured in beats. (TIF) [file pone.0263976.s001.tif]
